# Assessment of the Relationship between Body Mass Index and Gross Motor Development in Children

**Published:** 2017

**Authors:** Sepideh AMOUIAN, Zahra ABBASI SHAYE, Sakineh MOHAMMADIAN, Matin BAKHTIARI, Bahar PARSIANMEHR

**Affiliations:** 1 Neonatal and Children’s Health Research Center, Golestan University of Medical Sciences, Gorgan, Iran; 2 Pediatric Neurology Department, Faculty of Medicine, Golestan University of Medical Sciences, Gorgan, Iran; 3 Community Medicine Department, Mashhad University of Medical Science, Mashhad, Iran; 4 Medical student, Tehran University of Medical Sciences, Tehran, Iran

**Keywords:** Body mass index, Children, Gross motor development, ASQ, Denver II

## Abstract

**Objective:**

Obesity is a growing epidemic and public health problem in children. The purpose of this study was to determine the effect of body mass index (BMI) on the gross motor development.

**Materials & Methods:**

In this cross-sectional study conducted in 2012-13 in Gorgan, northern Iran, the gross motor development of 90 children 3-5 yr old in three groups of lean, normal and obese/overweight were evaluated by the ages and stages questionnaires (ASQ) and Denver 2 scale.

**Results:**

Totally, 90 children were enrolled and their developmental level was assessed with two ASQ and Denver II indices. The mean and standard deviation of the ASQ scores of the children was 53.11± 11.06 and based on Denver index, 9 children (10%) were at developmental delay status, 15 (16.7%) in the caution conditions, and 53 (58.9%) at normal developmental status. The developmental level was lower in obese/overweight group comparing with other groups according to both Denver and ASQ and there was a significant difference between obese/overweight group and normal group based in Denver and ASQ, respectively. There was no significant difference between underweight and normal and obese and underweight groups.

**Conclusion:**

Overweight and obesity could affect on the gross motor development.

## Introduction

Motor development is any change and progression in motor function resulting from a complex interplay of genetic and environmental factors. Motor development can be divided into two sections of gross motor and fine motor developments. The first is child’s ability to move around and to utilize all parts of his body to perform tasks like rolling, crawling, walking, running, and jumping. These skills usually require the use of all or several body parts simultaneously. 

Childhood obesity is a growing epidemic. The prevalence of childhood overweight and obesity is increasing dramatically all over the world (1-3). The global prevalence of childhood overweight in the Americas is greater than Europe (4).

About 14.4% of American children and adolescents are overweight or according to Body Mass Index (BMI) are at or above the 95th percentile (5). 

The health consequences of this epidemic are burst of adult diseases including hypertension, high cholesterol, type 2 diabetes, and cardiovascular disease among children. Some of the processes associated with poorer neurological function in obese adults have their origins in childhood (6). The frequency of the emergency and outpatient clinics utilization by overweight or obese children was more than underweight/healthy weight children were but was not for hospitalized cases (7).

Both motor activity and physical self-perception are affected by obesity (8). Significant weight loss may be considered an important means to upgrade the level of gross motor skill in overweight children (9).

BMI mediates the effect of income on children’s executive control and has negative implications for academic readiness, social competence, and behavioral adjustment (10, 11).

Obesity is a growing epidemic and a public health problem in children. Due to increase the prevalence of overweight and obesity and its impact on motor development and considering that previous studies have been conducted mainly on elementary school aged children; we decided to study and analyze the association between BMI and gross motor development in pre-school ( 3-5-yr-old) children.

## Materials & Methods

In this analytical cross-sectional study, 90 children of 3-5-yr-old from kindergartens in Gorgan, a northern city in Iran, were recruited by two-stage sampling in 2012-13 and were divided into three groups according to BMI: high, medium and low. Using comparison two proportions formula (12) and considering that 58% of overweight or obese children and 15% of children with normal BMI were rated below average GMQ (Gross Motor Quotient) score, the calculated sample size was 30 children for each group with total sample size of 90 (30 obese and overweight children, 30 children with normal BMI, 30 underweight children). Alpha error and power were defined 0.05 and 0.9, respectively. 

Ethics Committee of Golestan University of Medical Sciences approved the study. Parents were given the necessary information and were assured about the privacy of their personal data and informed consent was obtained. Parents of obese or overweight children were also given further explanations on the risks of obesity.

Inclusion criteria were restricted to healthy children registered in kindergartens and none of the children had a history of medical conditions such as chronic diseases, orthopedic problems, central or peripheral nervous system diseases that affect gross motor skills. 

These items were collected by questionnaire filled out by parents and examination done by the executive.

First, list of kindergartens in Gorgan with their addresses and geographic locations were prepared by Welfare Office and Department of Education. They were divided into North, South, East, and West groups based on their location in the city. Two kindergartens were selected randomly from each region. Parents and kindergartens’ officials were prepared with information and required measurements were done on children of these centers for 90 kids of 3-5-yr-old. Thirty of which had a normal BMI (between 5-85 percentile), 30 were obese or overweight (BMI at or above 85 percentile) and the rest were underweight or had a BMI below 5 percentile.

**BMI Measurement**

Children’s BMI were calculated based on standard growth charts of Centers for Disease Control and Prevention (CDC). The height and weight were measured by Seca scale and stadiometer (Germany) and obtained numbers using BMI formula (weight in kg divided by height in meters squared), were analyzed using BMI-for-age growth curves and percentiles were determined.

**Measuring gross motor development**

Children’s motor development was assessed according to ages and Stages Questionnaire (ASQ) as well as Denver II developmental Screening Test. ASQ is a standardized, valid and reliable questionnaire (13), filled out by parents and Denver II is a scale completed by the researcher. We used the valid and reliable Persian version of ASQ (14) and the Persian version of Denver II developmental Screening Test (15).

The ASQ are 19 parent report questionnaires that span the age range between 4 and 60 months. Questionnaire intervals include 4, 6, 8, 10, 12, 14, 16, 18, 20, 22, 24, 27, 30, 33, 36, 42, 48, 54, and 60 months of age. Each questionnaire composed of three sections: a brief set of demographic items, 30 questions on the infant’s or child’s development assessing five domains equally (communication, gross motor skills, fine motor skills, problem-solving, and personal-social skills), and seven open-ended questions eliciting parental concerns.

The choice of responses for each item is “yes,” “sometimes,” or “not yet,” receiving scores of 10, 5, and 0, respectively. Thus, ASQ scores of each child in each field can be between zero and 60. The test is graded according to the domain tested and compared with an empirically derived screening Cut off score (13, 15-19).

The Persian version of the ASQ has appropriate validity and reliability for screening developmental disorders in Iran (13). The ability to identify children with delay varied from 51% (4 months) to 90% (36 months), depending on the age at the time of assessment, with an overall sensitivity of 75% and a specificity of 81% to 92%, with an overall value of 86% (15, 20, 21). 

The Denver Developmental Screening Test, 2^nd^ edition is the most widely used test and is generally accepted worldwide because of its ease of use. It is highly reliable. This screening test is meant for children between 0 and 6 yr of age. It takes approximately 20 to 30 min to administer and score and involves a combination of formal testing, direct observation of the child, and eliciting possible parental concerns using a questionnaire. The test is composed of 125 items, divided into four sections: gross motor, fine motor/ adaptive, personal/social, or language skills. Each individual item is chosen appropriately according to the age of the child and scored objectively as a “pass,” “fail,” “refusal” (an uncooperative child), or “no opportunity” (if the item is not performed). A pass signifies that the child can perform a skill or task normally performed by 90% of the normative group younger than the child can.

A failed score is further graded as “delayed” if the child failed or refused a test item by 90% of younger children. Alternatively, it can be graded as a “caution” if the child failed or refused an item that 75% to 90% of younger children have already passed. The result of the test can be labeled as “normal,” “suspect,” or “untestable.” A normal result is produced when the child has no delays and a maximum of one caution, whereas a suspect is a child with two or more cautions or one or more definitive delays. Lastly, an “untestable” is a child who refuses to complete one or more tasks completed by 90% of a younger age group (15).

**Statistical analysis**

All analyses were performed with the use of SPSS software, ver. 11.5 (Chicago, IL, USA). Accumulation and distribution indices were used to describe data. 

Fisher and Chi-Square Tests (for qualitative variables), Kruskal–Wallis analysis (for quantitative variables) were used. We utilized Mann–Whitney Test to compare continuous quantitative variables. Normal distribution of variables was analyzed by Kolmogorov–Smirnov test. The significance level of less than 0.05 (P<0.05) and the confidence interval (CI) 95% were considered for all analyses.

## Results

Ninety children of 3-5-yr-old were categorized and analyzed in three 30-member groups of obese/ overweight, normal, and underweight in terms of gross motor development. The average age of children enrolled in our study was 47.58+/- 8.42 months, with a minimum and maximum age of 36, 60 months, respectively. Forty-seven (52/2%) were male and 43 (47.8%) were female. Table 1 shows the basic characteristics of the children listed in different groups by BMI.

**Table 1:** Basic characteristics of different BMI groups

***P*** **-value**	**Obese/overweight**	**Normal**	**Underweight**	**BMI**
0.25	46.43±8.86	49.67±7.92	46.63±8.83	Age (µ±SD)
0.56	Male 14(46.7) Female 16(53.3)	Male 18(60) Female 12(40)	Male 15(50) Female 15(50)	Gender (n(%))

Analysis showed that measure of agreement kappa between the results of ASQ and Denver was 0.82. In assessment of children development according to ASQ, we considered the adjusted age, which based on that children were divided into 5 groups: 36, 42, 48, 54, and 60 month-old.

Kruskal-Wallis test showed no statistically significant relationship between BMI and adjusted age groups (P=0.2). In Table 2, the mean, median and standard deviation of ASQ score, separated by adjusted age, are listed.

**Table 2:** ASQ and different adjusted-ages

**Standard deviation**	**Mean (median) ASQ score**	**Adjusted-age**
11.97	51.67(55)	36 month-old
12.65	51.36(60)	42 month-old
10.66	54.5(57)	48 month-old
10.04	54.25(60)	54 month-old
9.68	55(60)	60 month-old

Mean and standard deviation of ASQ score of male children was 55 +/- 9.78 with minimum of 25, maximum of 60, and median of 60. Mean ASQ score of female children was 51.05+/- 12.08 with minimum of 15, maximum of 60 and median of 55. Considering ASQ score, we found a statistically significant difference between male and female children (P=0.03)

but regarding BMI, no significant difference was seen between girls and boys in ASQ scores. Average and standard deviation of ASQ score in all participants was 53.11+/- 11.06. Table 3 shows ASQ score average in different BMI groups.

**Table 3:** ASQ score average in different BMI groups

**Standard deviation**	**ASQ average**	**BMI**
10.15	54.33	Underweight
5.81	57	Normal
13.93	48	Obese/overweight

We found a statistically significant difference among underweight, normal and obese/overweight children (P=0.01). Post Hoc analysis showed this difference was between normal BMI and obese/overweight groups (P=0.006). There was no significant difference between normal and underweight group (P=0.43) and between obese/overweight and underweight group (P=0.11).

Developmental status of children according to Denver scale was as follows: In male group; 4 (8.5%) participants were in delayed zone, 4 (8.5%) in border state, 31 children (66%) in normal zone, and 8 (17%) were in advanced status. 

In female children; 5 children (11.6%) were in the delayed condition, 11 children (25.6%) in border zone, 22 (51.2%) were normal and 5 children (11.6%) were in advanced zone.

Our analyses indicated that the developmental status of children based on Denver scale had no significant differences between both genders (P=0.14). It also had no statistically significant difference between both genders according to Denver scale among different BMI groups.

**Fig 1:**
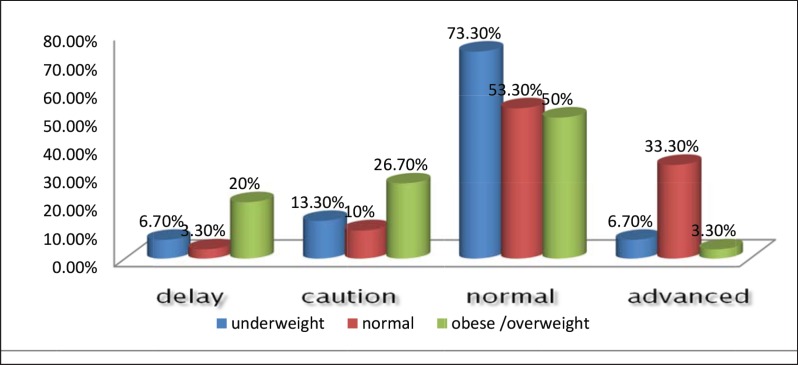
Frequency of children’s developmental status according to Denver in different BMI values

Among all children, 9 (10%) had delayed status based on Denver development index, 15 children (16.7%) were in border zone, 53 children (58.9%) were in normal developmental status, and 13 children (14.4%) were in advanced developmental state. Considering Denver development scale, we found a statistically significant difference among underweight, normal and obese/overweight children (P=0.002). This differences related to obese/overweight and normal children groups (P=0.004) and no statistically significant differences was observed between developmental status of children with normal weight and underweight (P=0.08) and between obese/overweight and underweight group (P=0.17). The frequency of children’s developmental status according to Denver in different BMI values is shown in Figure 1. The results of two tests and the BMI show in Table 4.

## Discussion

In our study, developmental level was assessed with two ASQ and Denver II indices. Among 90 children, the developmental level was lower in obese/overweight group comparing with other groups according to both Denver and ASQ and there was a significant difference between obese/overweight group and normal group, in agreement with other studies (4, 12, 22-27). However there was no significant difference between underweight and normal and obese and underweight groups, which does not agree with the other study (22). Possible explanations for this discrepancy are the number of the patients, increasing the sample size might lead us to a statistically significant and positive relationship between the level of development of underweight and normal children.

**Table 4:** Results of Denver, ASQ, and the BMI

**P-value**	**Denver**				***P*** **-value**	**ASQ**			**Failed**	
	**Delay**	**Caution**	**Normal**	**Advanced**		**Failed**	**Suspect**	**Passed**		
0.002	2(2.2)	4(4.4)	22(24.4)	2(2.2)	0.01	2(2.2)*	4(4.4)	24(26.7)	**Low**	**BMI**
	1(1.1)	3(3.3)	16(17.8)	10(11.1)		0	2(2.2)	28(31.1)	**Normal**	
	6(6.7)	8(8.9)	15(16.7)	1(1.1)		5(5.5)	6(6.7)	19(21.1)	**High**	

* : frequency (percent)

There was also no significant difference between males and females in our study, as in other studies (22, 26). 

However, there was difference between two genders (27, 28). No association with BMI in each of the four areas (cognition, language, fine and gross motor) of development in girls, but in boys, higher BMI was along with developmental delay in all four areas (28). 

However, this difference might be due to the large sample size.

There was a significant and inverse correlation between BMI and skills such as horizontal jumping, hopscotch, running, and dancing but there was no relationship between BMI and catching, shooting, and hitting a ball (29). In our study, we calculated motor developmental scores by summation of all scores, but in previously mentioned study, BMI was compared separately with each kind of movement. Only weight-dependent activities were correlated with BMI (29, 30). 

In this study, a statistically significant difference was detected between males and females in terms of ASQ score and boys gained a higher ASQ score comparing to girls but this difference was not observed in Denver II scale that might be due to detailed review of skills in ASQ index. The strength of our analysis includes simultaneous assessment motor development level with two famous ASQ and Denver II indices. 

**In conclusion, **obese and overweight children comparing to other children of their same age are at lower level of gross motor development. Increasing the sample size in further studies improves the accuracy of the results. Furthermore, providing a well-designed prospective study can strongly establish the causality between obesity and developmental delay. 

Determining the relationship between obesity and lower motor development level, we can warn parents about another negative aspect of their child obesity or overweight, therefore, they can prevent developmental delay in their child as well as other complications by controlling their diet and activity.
